# Intensified Stiffness and Photodynamic Provocation in a Collagen‐Based Composite Hydrogel Drive Chondrogenesis

**DOI:** 10.1002/advs.201900099

**Published:** 2019-07-15

**Authors:** Li Zheng, Sijia Liu, Xiaojing Cheng, Zainen Qin, Zhenhui Lu, Kun Zhang, Jinmin Zhao

**Affiliations:** ^1^ Guangxi Engineering Center in Biomedical Materials for Tissue and Organ Regeneration The First Affiliated Hospital of Guangxi Medical University No. 6 Shuangyong Road Nanning 530021 P. R. China; ^2^ Guangxi Collaborative Innovation Center for Biomedicine The First Affiliated Hospital of Guangxi Medical University No. 6 Shuangyong Road Nanning 530021 P. R. China; ^3^ Life Sciences Institute Guangxi Medical University No. 22 Shuangyong Road Nanning 530021 P. R. China; ^4^ Department of Medical Ultrasound Shanghai Tenth People's Hospital Tongji University School of Medicine 301 Yan‐chang‐zhong Road Shanghai 200072 P. R. China; ^5^ Department of Orthopaedics Trauma and Hand Surgery The First Affiliated Hospital of Guangxi Medical University No. 6 Shuangyong Road Nanning 530021 P. R. China

**Keywords:** cartilage repair, chondrogenesis, photodynamic provocation, reactive oxygen species, stiffness

## Abstract

Directed differentiation of bone‐marrow‐derived stem cells (BMSCs) toward chondrogenesis has served as a predominant method for cartilage repair but suffers from poor oriented differentiation tendency and low differentiation efficiency. To overcome these two obstacles, an injectable composite hydrogel that consists of collagen hydrogels serving as the scaffold support to accommodate BMSCs and cadmium selenide (CdSe) quantum dots (QDs) is constructed. The introduction of CdSe QDs considerably strengthens the stiffness of the collagen hydrogels via mutual crosslinking using a natural crosslinker (i.e., genipin), which simultaneously triggers photodynamic provocation (PDP) to produce reactive oxygen species (ROS). Experimental results demonstrate that the intensified stiffness and augmented ROS production can synergistically promote the proliferation of BMSCs, induce cartilage‐specific gene expression and increase secretion of glycosaminoglycan. As a result, this approach can facilitate the directed differentiation of BMSCs toward chondrogenesis and accelerate cartilage regeneration in cartilage defect repair, which routes through activation of the TGF‐β/SMAD and mTOR signaling pathways, respectively. Thus, this synergistic strategy based on increased stiffness and PDP‐mediated ROS production provides a general and instructive approach for developing alternative materials applicable for cartilage repair.

## 1 Introduction

In regenerative medicine, cartilage repair remains a challenging task, because articular cartilage exhibits a poor self‐repair ability after damages.^1^ In an attempt to develop an appropriate method for cartilage defect repair, stem cell therapy that usually seeds bone‐marrow‐derived stem cells (BMSCs) in scaffolds for enabling the lineage‐specific differentiation toward chondrogenesis has attracted increasing interest.^2^ However, due to inappropriate scaffold design, this strategy inevitably suffers from poor oriented differentiation tendency and low differentiation efficiency.^3^ Typically, the stiffness of scaffolds exerts robust influences on the directed differentiation of BMSCs, e.g., low stiffness can inhibit the chondrogenic differentiation of BMSCs, resulting in rapid degradation.^4^ As well, exogenous scaffolds are also known to trigger inflammation and produce immune repulsion‐derived side‐effects, further suppressing cartilage repair. Thus, to effectively direct the lineage‐specific BMSCs differentiation toward chondrogenesis, rationally designing scaffolds featuring appropriate chemical, topographic and mechanical properties is of great importance, yet to be achieved.

**Figure 1:**
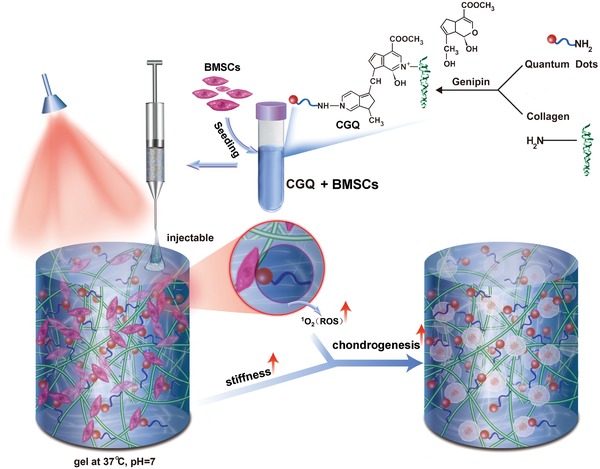
General schematic illustration of the fabrication process and implementation of collagen–genipin–quantum dot (CGQ) composite hydrogels.

To address these issues, we have established a combined strategy that integrates intensified stiffness with augmented reactive oxygen species (ROS) production to facilitate the chondrogenic differentiation of BMSCs as well as cartilage repair. To this end, an injectable collagen‐based composite hydrogel was designed and fabricated, wherein the collagen served as a scaffold to accommodate BMSCs and cadmium selenide (CdSe) quantum dots (QDs) via chemical crosslinking by a natural and nontoxic crosslinker (i.e., genipin),^5^ as indicated in **Figure** **1**. Genipin can guarantee excellent fluidity and injectability,^6^ reduce inflammatory responses,^7^ and simultaneously address the high toxicity and rapid gelation that traditional crosslinking agents encounter.^8^ Collagen features excellent injectability, inherent biocompatibility, favorable degradability and flexible environmental responsiveness.^9^ Herein, the used collagen type I is preferable than collagen type II, because it can repress inflammation and immune repulsion‐derived side‐effects,^10^ and facilitate chondrogenic differentiation of stem cells.^11^ To overcome its inherent low stiffness and poor fracture toughness,^12^ the crosslinking of collagen with biocompatible CdSe QDs by genipin was adopted to reinforce the stiffness of collagen,^13^ and benefit lineage‐specific differentiation of seeded BMSCs into chondrogenesis (Figure 1).^14^

As well, appropriate ROS levels are advantageous for cell growth and differentiation of BMSCs,^15^ and simultaneously fail to induce inflammation.^16^ Thus, CdSe QDs' introduction is also expected to further promote cell survival, proteoglycan secretion and cartilage repair by accelerating the lineage‐specific differentiation of BMSCs toward chondrogenesis,^17^ since CdSe QDs can serve as photosensitizers to produce ROS (e.g., singlet oxygen) via the photodynamic provocation (PDP).^18^ In vitro and in vivo experiments were carried out to confirm that the intensified stiffness and increased ROS production in this composite hydrogel can synergistically promote in vitro chondrogenesis and in vivo ectopic/orthotopic cartilage regeneration after seeding BMSCs.

## 2 Results and Discussion

### 2.1 Design and Fabrication of Collagen‐Based Composite Hydrogels

**Figure 2:**
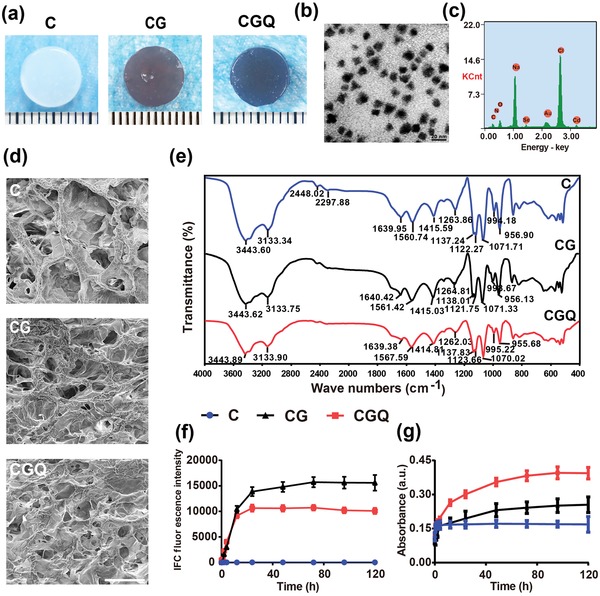
Characterizations of CGQ hydrogels. a) Photographs showing the formation of collagen‐based hydrogels. b) TEM image of QDs in CGQ nanocomposite, scale bar = 20 nm. c) EDS spectrum of CGQ hydrogels, and the *Y* axis indicates the signal intensity of the characteristic peak of related elements. d) SEM images of C, CG, and CGQ hydrogels (scale bar = 200 µm). e) FTIR spectra of C, CG, and CGQ composite hydrogels. f) Fluorescence intensity (excitation at 595 nm and emission at 630 nm) of C, CG, and CGQ hydrogels after crosslinking at different time points. g) The absorbance of collagen, CG and CGQ hydrogels at 595 nm after crosslinked at different time points. Data were expressed as the mean value ±standard deviation (SD), *n* = 5 (C = collagen, CG = collagen crosslinked with genipin, CGQ = collagen crosslinked with genipin and QDs).

The synthesis scheme of injectable collagen‐based composite hydrogels is shown in Figure 1, wherein collagen was chemically crosslinked with CdSe QDs via genipin crosslinker. Digital photos of collagen (C), collagen–genipin (CG), and collagen–genipin–QDs (CGQ) gels are shown in **Figure** **2**a. The dark green in CG and CGQ gels is attributed to the successful crosslinking of genipin with CdSe QDs and collagen through reacting with amines, suggesting that genipin can serve as an indicator of crosslinking based on its color change. Irregularly shaped CdSe QDs with an average diameter of 11.2 nm are found to be randomly oriented and uniformly distributed in CGQ and no size variation is observed (Figure 2b and Figure S1a, Supporting Information). Dynamic light scattering (DLS) also shows no evident size variation of QDs (Figure S2, Supporting Information). Much stronger characteristic peaks of selenium and cadmium elements in energy‐dispersive spectrometry (EDS) analysis confirm the successful linkage of more CdSe QDs in CGQ hydrogels (Figures 2c) in comparison to C and CG hydrogels (Figure S1b, Supporting Information). Noticeably, after crosslinking with genipin, the pore sizes of CG and CGQ hydrogels, which can be measured according to scanning electron microscopy (SEM) images,^19^ decreases from ≈100 to ≈50 µm (Figure 2d). Additionally, their Fourier transform infrared spectra (FTIR) further demonstrate the successful crosslinking of collagen with genipin and QDs in CGQ (Figure 2e).

In UV–vis spectra, a characteristic absorbance peak at 595 nm emerges in CG and CGQ gels due to the genipin crosslinking‐induced color change after 24 h incubation (Figure S3, Supporting Information). To further verify this result, the peak value at 630 nm in their photoluminescence spectra was recorded using a 595 nm laser as the excitation source. No obvious fluorescence signal harvested at 630 nm is observed in C hydrogels within 120 h (Figure 2f). In contrast, the fluorescence intensity in either CG or CGQ significantly increases as the incubation time proceeds and ultimately reaches a plateau after 12 h, confirming the characteristic peak of CG and CGQ hydrogels at 595 nm. Interestingly, the stronger UV–vis absorbance intensity in CG and CGQ hydrogels than that in C hydrogels at 595 nm further validates this point (Figure 2g). The minimum gelation time essential for completing the gelation process was obtained in Table S1 (Supporting Information), wherein the three hydrogels can keep fluid at 4 °C for over 22 h, and remaining fluid for over 7 min even at 37 °C, which sufficiently guarantees the injectability of these hydrogels.

### 2.2 Evaluations on Structural Properties of CGQ

**Figure 3:**
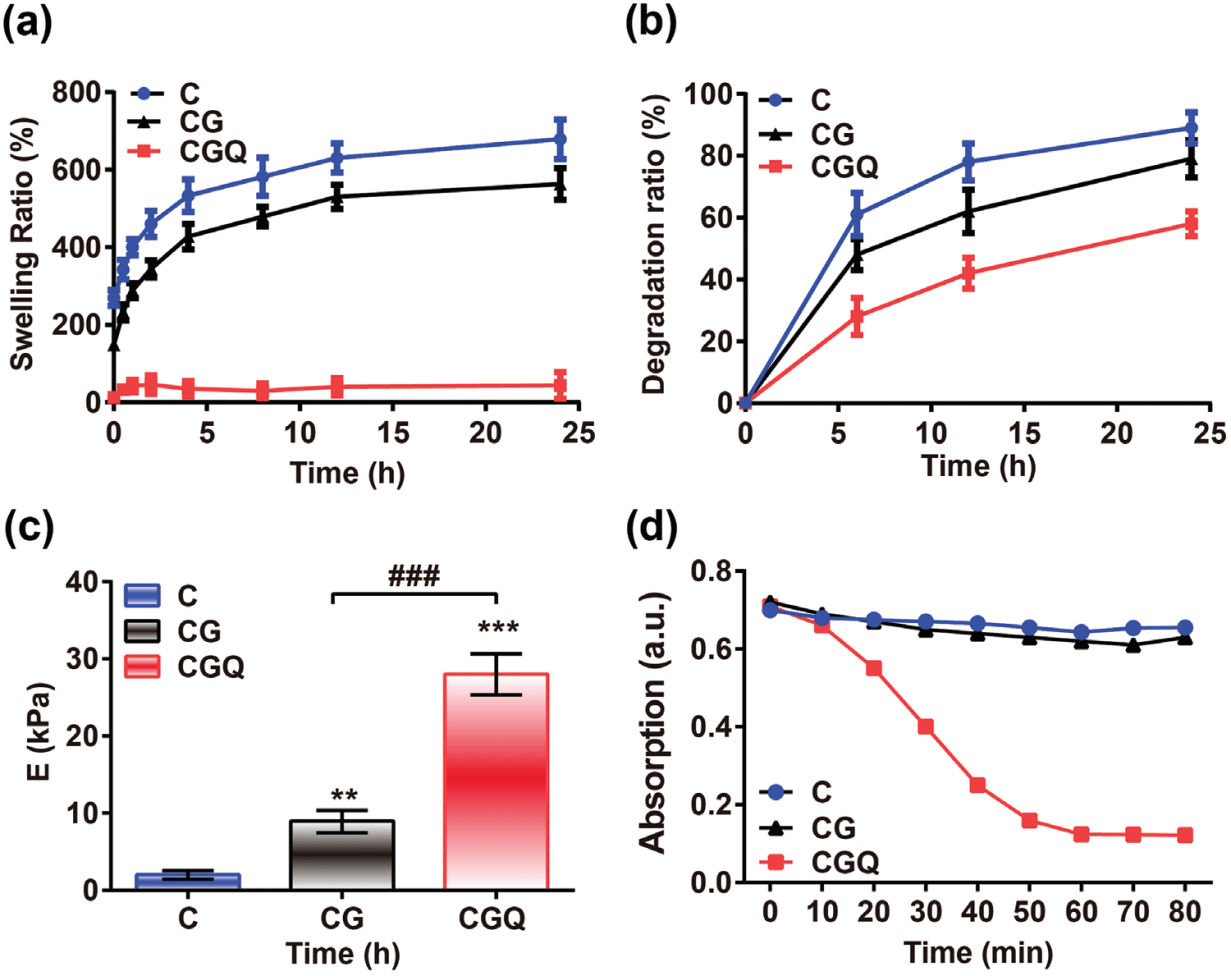
Mechanical and photodynamic characterizations of CGQ hydrogels. a–c) The swelling ratio (a), degradation rate at a type I collagenase concentration of 100 µg mL^−1^ (b) and Young's modulus obtained at 10–20% linear curve (c) of C, CG, and CGQ hydrogels. d) Absorption value of DPBF probe after incubation with C, CG, or CGQ for different irradiation durations. Data are expressed as mean ± SD (*n* = 6); ** indicates *p* < 0.01; *** and ^###^ indicate *p* < 0.001 (C = collagen, CG = collagen crosslinked with genipin, CGQ = collagen crosslinked with genipin and QDs).

Swelling ratio that is a routine concern for hydrogel scaffolds was investigated. Compared with CG hydrogels, the chelation of CdSe QDs in CGQ via covalently anchoring by the genipin crosslinker endows CGQ with a robust anti‐swelling property (**Figure** **3**a). The degradation rate of scaffolds is another concern in tissue engineering, and an ideal degradation rate, in principle, is approximately consistent with the rate of tissue regeneration. Interestingly, the incorporations of QDs and genipin via mesh crosslinking with collagen endow CGQ with improved mechanical property and stronger resistance to degradation catalyzed by type I collagenase than C and CG (Figure 3b), which will be beneficial for rendering CGQ degradation matched with cartilage regeneration. To comprehensively explore their degradation behaviors, in vitro degradation profiling at a concentration of 10 ng mL^−1^ that is close to that in serum in vivo shows that the degradation ratios of all the three gels approach 100% after 60 days (Figure S4, Supporting Information).

Scaffold stiffness is known to exert a significant influence on the differentiation of stem cells.^20^ Despite being a common scaffold in cartilage tissue engineering, collagen‐based hydrogels still suffer from unmatched mechanical properties and rapid degradation that are unfavorable for chondrogenesis. Given that chemically doped nanoparticles and crosslinking can improve the stiffness and stability of hydrogels to resist denaturation and enzymatic degradation,^14^ CdSe QDs and biocompatible genipin crosslinkers are also anticipated to reinforce the stiffness of CGQ and drive lineage‐specific differentiation of BMSCs toward chondrogenesis. Indeed, contributed by the robust crosslinking between collagen scaffolds and CdSe QDs by genipin, the stiffness of CGQ hydrogel (28.7 ± 2.6 kPa) is considerably improved (Figure 3c) with 14‐fold and threefold larger than that of C (1.9 ± 0.3 kPa) and CG hydrogels (9.53 ± 2.2 kPa), respectively. Although the equilibrium modulus (28.7 ± 2.6 kPa) of CGQ hydrogel is much lower than that of native cartilage (1–10 MPa), the soft CGQ scaffold is expected to enable cells to sense stiffness change and benefit cartilage regeneration, since most work has shown a stiffness sensing mechanism for evaluating cell responses on matrices much softer than native cartilage.^21^

### 2.3 ROS Production

Furthermore, the ability of QDs in CGQ hydrogels to produce ROS was investigated since appropriate ROS level is also responsible for regulating BMSCs differentiation.^15^ CdSe QDs have been well documented to generate singlet oxygen via the PDP process.^18^ Herein, 1‐ 3‐diphenylisobenzofuran (DPBF) as the sensitizer of singlet oxygen was used to monitor the in vitro ROS production in CGQ hydrogels upon exposure to 808 nm laser irradiation.^22^ As expected, the production level of singlet oxygen representing ROS in CGQ hydrogels is much higher than that in C and CG hydrogels, as demonstrated by the drastic decline of the absorbance intensity of DPBF in the CGQ group (Figure 3d). This result indicates that CdSe QDs embedded in CGQ indeed generated singlet oxygen via the 808 nm laser‐activated PDP to oxidize DPBF.

**Figure 4:**
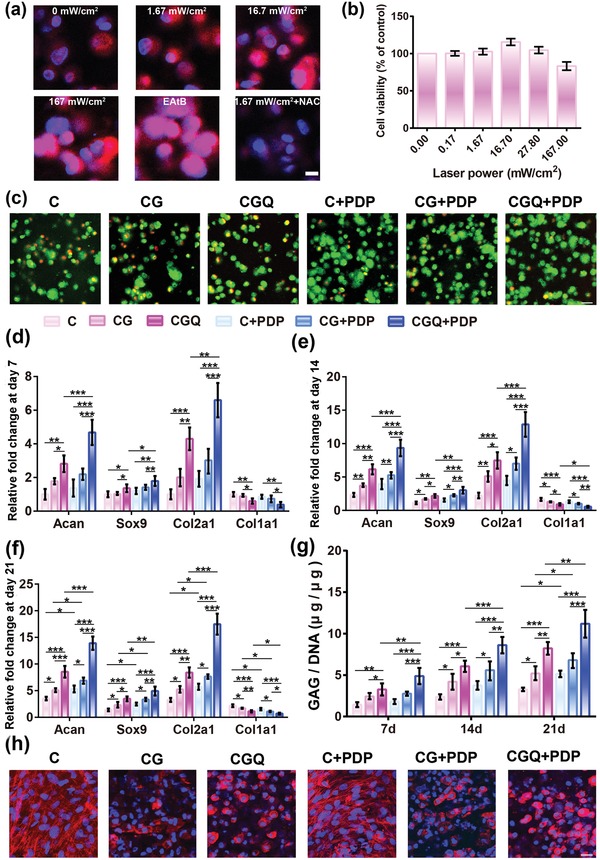
In vitro chondrogenesis of BMSCs induced by intensified stiffness and ROS production in CGQ. a) Laser confocal microscopy images of cells embedded in CGQ after treatment with PDP at various laser power densities for 3 min, wherein ROS production was assessed with DHE probe (pink color) and stronger pink fluorescence signal means more ROS; NAC (a ROS scavenger) and EAtB (1 × 10^−3^ m Elesclomol+100 × 10^−3^ m AAPH+100 × 10^−3^ m tBHP, the ROS inducer) were used; scale bar = 20 µm. b) In vitro viabilities of BMSCs cultured in CGQ scaffold after irradiation with different laser fluences for 3 min. c) Cell viability of BMSCs cultured in the different scaffolds (i.e., C, CG, and CGQ) with or without PDP for a period of 21 days (scale bar = 30 µm). d–f) mRNA expression and g) GAG content of BMSCs after different treatments for 7 days (d), 14 days (e), and 21 days (f). h) Cell skeleton staining of the cells cultured in the scaffolds with or without PDP for 21 days (scale bar = 100 µm). Mean ± SD, *n* = 5; *, ** and *** indicates *p* < 0.05, 0.01, and 0.001, respectively (C = collagen + BMSCs, CG = collagen crosslinked with genipin + BMSCs, CGQ = collagen crosslinked with genipin and QDs + BMSCs, C + PDP = collagen + BMSCs + irradiation with an 808 nm laser at fluence of 3 J cm^−2^ for 3 min, CG + PDP = CG scaffold + BMSCs + irradiation with an 808 nm laser at fluence of 3 J cm^−2^ for 3 min, CGQ + PDP = CGQ scaffold + BMSCs + irradiation with an 808 nm laser at fluence of 3 J cm^−2^ for 3 min).

Furthermore, intracellular ROS production was evaluated using a dihydroethidium (DHE) probe. A larger power density brings about a stronger fluorescence signal, suggesting more ROS production in cells (**Figure** **4**a), since the pink fluorescence intensity positively correlates with the level of ROS production. In particular, upon exposure to 167 mW cm^−2^, the fluorescence intensity is approximately identical to that in the ROS inducer (i.e., EAtB)‐treated group, suggesting the same ROS level . However, the signal was extinguished after adding the ROS scavenger, i.e., *N*‐acetyl cysteine (NAC), which decreased ROS production despite an identical PDP.^23^ These results sufficiently validate ROS production via the PDP in CGQ hydrogels, which enables the facilitated differentiation of BMSCs toward chondrogenesis. Afterward, in vitro safety of CGQ‐mediated ROS was evaluated to determine the optimal power density under which the viability can reach the largest value. As indicated in Figure 4b, CGQ treatment with 808 nm laser at a power density of 16.7 mW cm^−2^ corresponding to 3 J cm^−2^ gives birth to the largest cell viability, denoting that the optimal in vitro laser fluence is 3 J cm^−2^.

### 2.4 Improved In Vitro Proliferation and Differentiation of BMSCs Using CGQ in Combination with Laser Irradiation

It is reported that increased ROS production may intensify cell proliferation in CGQ.^24^ Indeed, after incubation with CGQ for 21 days, BMSCs in the CGQ+PDP group attain the highest proliferation rate, as evidenced by the qualitative observation (Figure 4c) and quantification analysis (Figure S5, Supporting Information). Besides promoting BMSC proliferation, ROS can also promote differentiation and proteoglycan secretion of BMSCs,^25^ thus yielding a hypothesis that increased ROS together with intensified stiffness may produce a synergistic effect in promoting BMSC differentiation. To validate this hypothesis, quantitative real‐time polymerase chain reaction (qRT‐PCR) was used to analyze cartilage‐specific gene expression including *Acan, Sox9, Col2a1*, and *Col1a1* (Table S2, Supporting Information) which regulate the differentiation of BMSCs.^26^ In all periods examined, higher expression levels of cartilage‐specific genes (i.e., *Acan*, *Sox9*, and *Col2a1*) in BMSC‐seeded CGQ hydrogels are observed when compared with that in C or CG hydrogels (Figure 4d–f). This result validates that the intensified stiffness induced by weaved QDs and crosslinker in CGQ is responsible for the pro‐chondrogenic outcome. Furthermore, upon exposure to laser irradiation, ROS arising from QD‐mediated PDP in CGQ+PDP enables CGQ hydrogels to induce the highest expression of *Acan*, *Sox9*, and *Col2a1* regardless of the length of incubation time (Figure 4d–f). Concurrently, the intensified stiffness and ROS production in CGQ+PDP result in significant down‐regulation of a fibrocartilage marker (i.e., *Col1a1*).^26^ These intriguing results indicate that the synergistic effect of appropriate ROS and intensified stiffness will facilitate the BMSCs differentiation and cartilage repair through the upregulations of *Acan*, *Sox9*, and *Col2a1* and downregulation of *Col1a1*.

Next, the secretion of GAG as a primary component in the extracellular matrix (ECM) of cartilage was monitored to indirectly evaluate the regeneration degree of ectopic cartilage. Consistent with the ranking order of ROS and stiffness, the CGQ+PDP group results in the largest CGA content, and a continuous increase over time is also observed but not in C and CG hydrogels (Figure 4g). To visualize the differentiation of BMSCs in CGQ+PDP, the cytoskeletal morphology of BMSCs after phalloidin (PI)/DAPI co‐staining was evaluated using laser confocal scanning microscopy (LCSM). Elongation of cells with F‐actin fibers in the cytoplasm that is a typical characteristic of undifferentiated stem cells is observed in all groups (Figure 4h). In contrast, round‐shaped chondrocytes that severely lose stress fibers are mainly present in CGQ hydrogels, suggesting the successful differentiation of BMSCs in the CGQ group. Upon further laser irradiation, PDP‐mediated ROS trigger more aggregates of round cells, further enhancing the differentiation of BMSCs into chondrocytes.

### 2.5 In Vivo Hyaline Cartilage Regeneration Based on Intensified Stiffness and ROS Production in CGQ in a Noncartilaginous Environment

**Figure 5:**
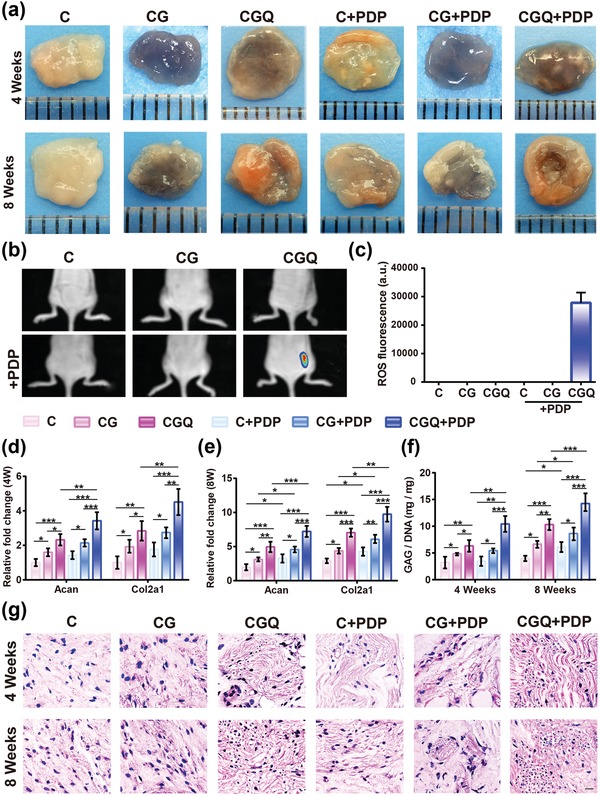
In vivo evaluations on BMSCs differentiation toward chondrogenesis in the noncartilaginous environment based on the intensified stiffness and ROS production in CGQ. a) Macroscopic observation of subcutaneously implanted tissues in nude mice with or without PDP for 4 and 8 weeks. b) In vivo fluorescence imaging showing ROS generation in the mice implanted with four composites subcutaneously with or without PDP. c) The fluorescence intensity based on data from (b). d,e) Expression levels of chondrogenesis‐specific genes, i.e., *Acan* and *Col2a1*, in regenerated tissues after subcutaneous implantation in nude mice for 4 weeks (d) and 8 weeks (e). f) GAG content in regenerated tissues with subcutaneous implantation in nude mice with or without PDP for 4 and 8 weeks (normalized to the DNA content). Mean±SD, n = 6; *, ** and *** indicate *p* < 0.05, 0.01, and 0.001, respectively. g) Hematoxylin and eosin (HE) staining for tissues with subcutaneous implantation in nude mice with or without PDP for 4 and 8 weeks (scale bar = 40 µm). Note: C = collagen + BMSCs, CG = collagen crosslinked with genipin + BMSCs, CGQ = collagen crosslinked with genipin and QDs + BMSCs, C + PDP = collagen + BMSCs + irradiation with an 808 nm laser at fluence of 3 J cm^−2^ for 3 min, CG + PDP = CG scaffold + BMSCs + irradiation with an 808 nm laser at fluence of 3 J cm^−2^ for 3 min, CGQ + PDP = CGQ scaffold + BMSCs + irradiation with an 808 nm laser at fluence of 3 J cm^−2^ for 3 min.

To validate the synergistic effect in vivo, BMSCs seeded in C, CG, and CGQ hydrogels were subcutaneously injected into nude mice, and in vivo ectopic cartilage photos were captured after 4 and 8 weeks post‐transplantation, respectively. Comparing to C and CG hydrogels with loose structures, CGQ yields compact tissues with elasticity similar to native articular cartilage (**Figure** **5**a), validating the ability of intensified stiffness in CGQ scaffolds to drive cartilage regeneration. Upon exposure to laser irradiation, QDs in CGQ responded to laser and generated ROS which further promoted BMSCs differentiation into chondrocytes, resulting in massive neotissue formation in CGQ+PDP (Figure 5a). Taken together, the in vivo chondrogenesis results sufficiently demonstrate that the intensified stiffness and augmented ROS production in CGQ+PDP indeed promoted BMSCs differentiation into chondrocytes and gave birth to new and compact cartilage tissues. Interestingly, the dark color representing CG and CGQ is still detectable after the 4th week, but fades away after the 8th week, indicating the concurrent emergence of nascent cartilage‐like tissues and scaffolds' degradation.

To comprehensively understand the roles of intensified stiffness and ROS production in facilitating cartilage regeneration, deep principle was explored. ROS levels in the subcutaneously implanted noncartilaginous environments that received different treatments were first examined. As shown in Figure 5b,c, evident fluorescence signal is observed in the group of CGQ combining with laser (i.e., CGQ+PDP), but not in other groups, indicating that in vivo CdSe QD‐mediated ROS production occurred only in the CGQ+PDP group. Similar to in vitro results, more expression of *Acan* and *Col2a1* was induced by intensified stiffness in BMSC‐seeded CGQ hydrogels, which was further strengthened by ROS after laser irradiation (Figure 5d,e). The largest GAG secretion in the transplanted tissues also occurred in the CGQ+PDP group (Figure 5f). These intriguing results further support that intensified stiffness and ROS production cooperatively contributed to the accelerated chondrogenesis.

Furthermore, pathological examination by HE staining shows fibrocartilage‐like tissues in C or CG hydrogels, but spherical chondrocyte cells embedded in the lacuna that are the typical representative of hyaline cartilage phenotype are found in CGQ alone or CGQ combined with laser irradiation (Figure 5g). This phenomenon definitely indicates the occurrences of chondrogenesis differentiation of embedded BMSCs and cartilage regeneration in CGQ alone or CGQ+PDP. The complete disappearance of residual scaffolds represented by blank (i.e., no staining) after 8 weeks post‐injection suggests an excellent degradation rate in vivo that is consistent with the cartilage regeneration rate, akin to in vitro result (Figure S4, Supporting Information). In addition, CGQ in combination with PDP demonstrates an excellent safety profile as no obvious body weight loss of mice is observed in any of the groups (Figure S6, Supporting Information).

### 2.6 Accelerated Cartilage Regeneration in Defects using CGQ in Combination with Laser Irradiation (i.e., CGQ+PDP)

**Figure 6:**
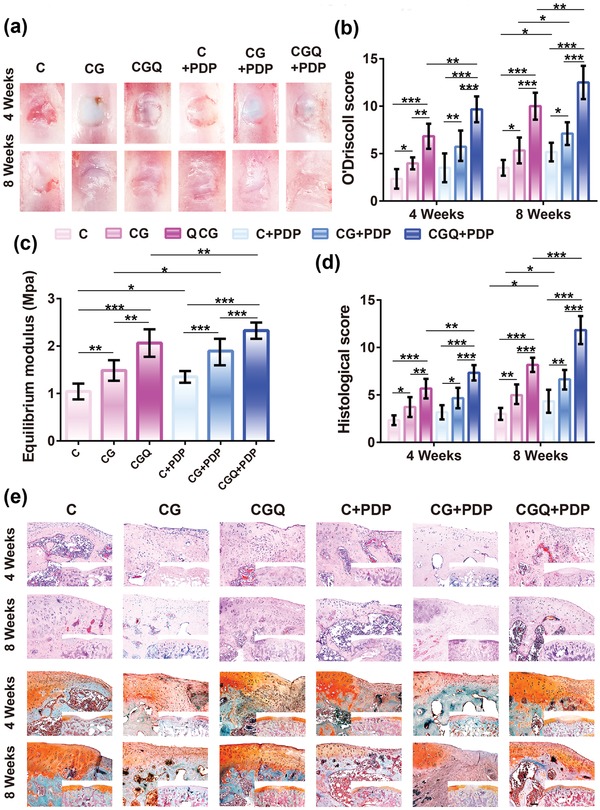
In vivo cartilage regeneration in defects after implanting BMSC‐seeded CGQ scaffolds. a,b) Gross macroscopic observation (a) and O'Driscoll score (b) of engineered cartilage tissues subjected to different treatments after 4 and 8 weeks, respectively. c) Mechanical property evaluation of the engineered cartilage tissues subjected to different post‐treatments after 8 weeks. d) Histological score of the engineered cartilage tissues subjected to different post‐treatments after 4 and 8 weeks, respectively. e) HE (top) and Safranin O (bottom) staining of the engineered cartilage tissues subjected to different post‐treatments (scale bar = 200 µm). Mean ± SD (*n* = 6); *, ** and *** indicates *p* < 0.05, 0.01, and 0.001, respectively. (C = collagen + BMSCs, CG = collagen crosslinked with genipin + BMSCs, CGQ = collagen crosslinked with genipin and QDs + BMSCs, C + PDP = collagen + BMSCs + irradiation with an 808 nm laser at fluence of 3 J cm^−2^ for 3 min, CG + PDP = CG scaffold + BMSCs + irradiation with an 808 nm laser at fluence of 3 J cm^−2^ for 3 min, CGQ + PDP = CGQ scaffold + BMSCs + irradiation with an 808 nm laser at fluence of 3 J cm^−2^ for 3 min).

Inspired by above success in facilitating BMSCs differentiation into a chondrocyte phenotype and accelerating cartilage regeneration, such a synergistic effect for cartilage defect repair by regenerating cartilage‐like tissues was explored. Identical to aforementioned in vitro results, the laser setting, i.e., 3.0 J cm^−2^ (16.6 mW cm^−2^) for 3 min, is also determined as the optimal dose of laser fluence in this in vivo evaluation (Figure S7, Supporting Information). Digital photos show that the boundaries between uneven reparative tissues and original cartilage in C, CG, and CGQ hydrogels are distinguishable after 4 weeks post‐transplantation. However, the reparative tissues in the C and CG groups with or without PDP are loose and fibrous, while the regenerated tissues in the CGQ group are more compact and interconnected with the adjoining cartilage especially after exposure to laser irradiation (i.e., CGQ+PDP) (**Figure** **6**a). After 8 weeks, the regenerated tissues in the C and CG groups are still loose and nonintegrated with the adjacent normal cartilage. In contrast, the newborn tissues in defects treated with CGQ evolve into smooth and well‐integrated ones with surrounding tissues (Figure 6a). Interestingly, the boundary between regenerated tissues and adjacent cartilage in CGQ+PDP vanishes, suggesting no obvious difference between repaired cartilage and adjacent cartilage.

Noticeably, two characteristics of inflammation or synovitis, i.e., osteophytes formation and cartilage erosion, are not evident in all groups (Figure 6a), which suggests that a moderate ROS production in CGQ+PDP is insufficient to induce inflammation that may damage articular cartilage. This result is consistent with previous reports that the dominant skeleton (i.e., collagen) in CGQ as a normal composition of human body fails to induce inflammation.^27^ We further used O'Driscoll scoring to directly assesses the progress of repaired cartilage tissues. CGQ+PDP harvests the highest scores (Figure 6b), further confirming that CGQ+PDP performed the best in cartilage repair.

As a load‐bearing material of diarthrodial joints, articular cartilage can absorb mechanical shocks and distribute high joint loads more evenly, simultaneously maintaining minimal friction and wear.^28^ Thus, detecting the equilibrium modulus of regenerated cartilage tissues is of significance. Herein, the stiffness of regenerated cartilage tissues after 8 weeks post‐transplantation was evaluated using equilibrium modulus. Identical to the ranking order of aforementioned O'Driscoll scoring, the stiffness ranking of regenerated tissues in different groups follows the order: C < CG < CGQ, and 96% and 32% increases in comparison to C and CG, respectively, are obtained in CGQ, suggesting that the intensified stiffness of CGQ scaffold caused more robust regenerated cartilage. Furthermore, the synergistic effect involving intensified stiffness and ROS production in CGQ+PDP enables the regenerated tissues to acquire the largest stiffness with an increase of compression modulus by 124% (Figure 6c). Notably, the stiffness value (above 2 MPa) of regenerated cartilage tissues in either CGQ alone or CGQ+PDP groups after 8 week post‐implantation is within the stiffness window (1–10 MPa) of native cartilage, which suggests that the regenerated tissues can perform the normal function of native cartilage. These results demonstrate the validity of this general combined approach for guiding the design of cartilage‐repairing materials.

Histological evaluations by HE and safranin‐O staining show that cartilage defects are filled with nascent tissues in all the groups (Figure 6e). In C and CG, fibrous and immature tissues with a loose boundary that displays a discontinuous connection with the surrounding cartilage are almost negatively stained by safranin‐O and dominant in defects after 4 weeks post‐operation. Subsequently, they further rounded into fibrocartilage‐like tissues after 8 weeks. In contrast, fibrocartilage‐like tissues are first observed in defects treated with CGQ hydrogels after 4 weeks post‐operation, but subsequently evolved into hyaline cartilage tissues that are positively stained by safranin‐O after 8 weeks. Concurrently, the regenerated cartilage tissues exhibit a tight boundary adjacent to cartilage tissues. In particular, after exposure to laser irradiation, the regenerated tissues that are positively stained by intense red color in CGQ+PDP manifest no evident difference from the surrounding normal tissues. Quantitatively, the regenerated tissues in CGQ+PDP receive the highest histological scoring (Figure 6d), indicating the accelerated healing of cartilage defects due to the intensified stiffness and ROS production. Intriguingly, the ranking order of histological scoring in different groups is consistent with that of aforementioned O'Driscoll scoring. Noticeably, pathological examination after H&E staining also fails to detect inflammatory cells, which further validates no inflammation in all the groups and indicates the biosafety of CGG scaffolds and moderate ROS. As well, the complete disappearance of scaffolds that is represented by no blank in both HE and safranin‐O staining after 8 weeks post‐injection further demonstrates the excellent in vivo degradation of collagen‐based scaffolds. More significantly, the in vivo degradation rate of CGQ scaffold completely matches the time frame of cartilage formation.

More intense positive immunohistochemical staining in the CGQ+PDP group indicates more type II collagen in the regenerated tissues (Figure S8, Supporting Information). In contrast, the mostly negative staining in the C and CG groups after either 4 or 8 weeks post‐transplantation suggests the absence of hyaline‐like cartilage, which is in accordance with the results in above experiments. Additionally, the histological slices of engineered cartilage tissues after 4 or 8 weeks post‐implantation were detected by LCSM, and no fluorescence signal of CdSe QDs suggests neglectable internalization by cells (Figure S9, Supporting Information).

### 2.7 Signaling Pathways Activated in Chondrogenic Differentiation Promoted by Intensified Stiffness and ROS Production

**Figure 7:**
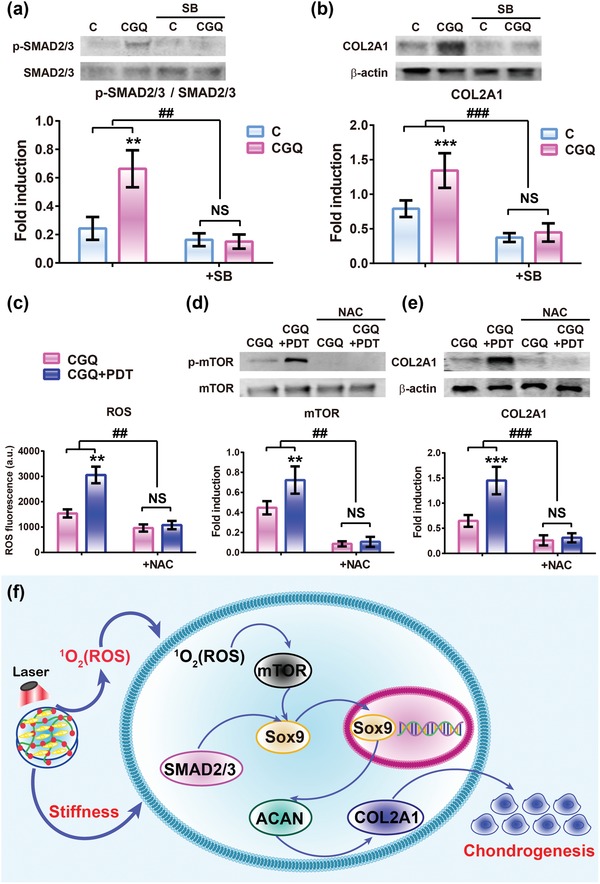
Signaling pathway exploration of chondrogenic differentiation promoted by intensified stiffness and augmented ROS production in CGQ. a,b) Expression levels of SMAD2/3, phosphorylated SMAD2/3 (p‐SMAD2/3) (a) and COL2A1 proteins (b) in cells cultured in C and CGQ scaffolds in vitro for 14 days with or without SB (SB43154). c–e) ROS generation (c), expression levels of mTOR, phosphorylated mTOR (p‐mTOR) (d) and COL2A1 (e) proteins in the cells encapsulated in CGQ scaffold in vitro with or without PDP for 14 days (NAC was used). f) Schematic of the associated signaling pathways involved in mechanotransduction and laser irradiation. Mean ± SD, *n* = 5; ** and ## indicate *p* < 0.01; *** and ### indicate *p* < 0.001 (C = collagen + BMSCs, CGQ = collagen crosslinked with genipin and QDs + BMSCs, C + PDP = collagen + BMSCs + irradiation with an 808 nm laser at fluence of 3 J cm^−2^ for 3 min, CGQ + PDP = CGQ scaffold + BMSCs + irradiation with an 808 nm laser at fluence of 3 J cm^−2^ for 3 min).

Next, we investigated the signaling pathways associated with BMSCs differentiation into chondrogenesis and cartilage repair promoted by intensified stiffness and ROS production. The influence of intensified stiffness on TGF‐β/SMAD signaling pathway associated with mechanotransduction,^29^ was first investigated, since this pathway may regulate cartilage maintenance during embryonic evolution.^30^ In this pathway, SMAD2 and SMAD3 are downstream transcription factors that are usually phosphorylated in chondrocytes deriving from articular cartilage via mechanotransduction‐mediated TGF‐β1 or β3 ligand/receptor binding.^31^ Thus, it is anticipated that the intensified stiffness in CGQ might be able to increase the phosphorylation of SMAD 2/3 through activating the TGF‐β/SMAD signaling pathway. As expected, the expression levels of SMAD2/3 and phosphorylated SMAD2/3 (p‐SMAD2/3) in BMSC cells treated with CGQ hydrogels are considerably enhanced (>2.6‐fold) compared with that treated with C hydrogel due to the augmented compression modulus (**Figure** **7**a). Moreover, a specific inhibitor of TGF‐β signaling pathway, i.e., SB431542,^32^ was applied to significantly reduce the levels of SMAD2/3 and p‐SMAD2/3. Similarly, the expression level of COL2A1 that is another effector of chondrogenesis is also increased by 1.7‐fold in the CGQ group, but effectively suppressed by SB431542 (Figure 7b). These results validate the involvement of TGF‐β/SMAD signaling pathway in BMSCs chondrogenesis promoted by intensified stiffness in CGQ and suggest that matrix stiffness tuning can serve as an effective approach to augment chondrogenesis.

As another pivotal factor, ROS were also demonstrated to facilitate the chondrogenic differentiation of BMSCs and cartilage repair in aforementioned experiments. It has been well documented that ROS can mediate cellular alterations of crucial second messengers to promote differentiation through activating mTOR signaling pathway that is critical to chondrogenesis.^33^ Thus, evaluations on ROS‐mediated mTOR signaling pathway were undertaken, wherein mTOR phosphorylation and COL2A1 expression were highlighted. In Figure 7c, laser irradiation induces marked abundant ROS production in CGQ, which was accompanied by increased mTOR phosphorylation and COL2A1 expression in BMSCs embedded in CGQ+PDP by 60.2% and 120.5%, respectively, compared with CGQ alone (Figure 7d,e). After adding a ROS scavenger (e.g., NAC),^23^ the expression of mTOR phosphorylation and COL2A1 are tremendously suppressed and no obvious difference is observed between CGQ and CGQ+PDP. These results confirm the role of ROS in promoting chondrogenesis via activating mTOR signaling pathway.

Given the above results, we proposed a model that depicts the upstream and downstream signaling events in chondrogenesis associated with stiffness and ROS (Figure 7f). In detail, laser irradiation can trigger BMSC‐seeded CGQ scaffolds to produce ROS via the QD‐mediated PDP process, which activates the mTOR signaling pathway to promote more expression of a cartilage‐specific gene, i.e., Sox9. On the other hand, the intensified stiffness induced by crosslinking with QDs and genipin in the CGQ scaffold can exert considerable influences on the TGF‐β/SMAD signaling pathway to further induce Sox9 expression. The activated Sox9 subsequently activates the other two downstream cartilage‐specific genes, i.e., ACAN and COL2A1 in sequence, facilitating chondrogenesis. Therefore, enhancing stiffness and ROS production can serve as a unified method to achieve highly efficient cartilage repair by driving BMSCs differentiation. As well, the flexible hydrogels can be engineered into various shapes objective to irregular‐shaped defects (Figure S10, Supporting Information).

## 3 Conclusion

In summary, an injectable collagen‐based composite hydrogel (CGQ) was obtained using collagen as the platform for carrying BMSCs and crosslinking CdSe QDs. This composite hydrogel exhibits appropriate biodegradability and excellent biocompatibility. The crosslinking and QDs introduction made the stiffness of CGQ considerably improved, and CGQ could give rise to increased ROS in the presence of laser irradiation via the QD‐mediated PDP process. A synergistic effect involving intensified stiffness and augmented ROS production has been demonstrated to promote more expression of cartilage‐specific genes and accelerate BMSCs differentiation into chondrocytes both in vitro and in vivo, enabling cartilage repair in cartilage defects. More significantly, the two means have been demonstrated to engage different pathways to regulate sequential expression of cartilage‐specific genes, e.g., intensified stiffness activates the TGF‐β/SMAD signaling pathway, while ROS production correlates with mTOR signaling pathway. Collectively, this synergistic strategy and the corresponding composite hydrogels lay a solid foundation to highly efficient cartilage regeneration for future clinical application and offer a new avenue to guide the design of new scaffolds capable of cartilage repair.

## 4 Experimental Section

Materials, methods, all experimental details, and procedures are included in the Supporting Information.

## Conflict of Interest

The authors declare no conflict of interest.

## Supporting information

Supplementary
