# Supplementary material for: Intensified Stiffness and Photodynamic Provocation in a Collagen‐Based Composite Hydrogel Drive Chondrogenesis
Source: Adv Sci (Weinh). 2019 Jul 15;6(16):1900099. doi: 10.1002/advs.201900099 (PMC6702628; doi:10.1002/advs.201900099)
Supplement: Supplementary file 1 — Supplementary [file ADVS-6-1900099-s001.pdf]

Copyright WILEY-VCH Verlag GmbH & Co. KGaA, 69469 Weinheim, Germany, 2019.

ADVANCED SCIENCE
Open Access

Supporting Information

for *Adv. Sci.,* DOI: 10.1002/advs.201900099

Intensified Stiffness and Photodynamic Provocation in a Collagen-Based Composite Hydrogel Drive Chondrogenesis

Li Zheng, Sijia Liu, Xiaojing Cheng, Zainen Qin, Zhenhui Lu, Kun Zhang,* and Jinmin Zhao**

Intensified Stiffness and Photodynamic Provocation in Collagen-based Composite Hydrogel Drive Chondrogenesis

Li Zheng,[#] Sijia Liu,[#] Xiaojing Cheng,[#] Zainen Qin, Zhenhui Lu, Kun Zhang* and Jinmin Zhao**

Supplementary experimental sections

Characterization

A drop of QDs or CGQ solution was placed onto a copper grill covered by a polymer film and then dried at room temperature. Transmission electron microscopy (TEM) images were acquired on a transmission electron microscope operated at 100 kV. The size distributions of QDs in aqueous solution or CGQ were obtained by DLS (ZetaSizer Nano ZS, Malvern Instruments Ltd., Worcestershire, UK). The morphology and the pore size of collagen, CG and CGQ hydrogels were assessed by SEM (TESCAN, USA) at an accelerating voltage of 15 kV, before which the hydrogels were freeze-dried and then gold-coated to avoid the charging effect. FTIR spectra of the collagen, CG and CGQ scaffolds were determined on a Spectrum 100 spectrometer in the spectral range of 4000-400 cm^{-1}. The prepared materials were frozen in liquid nitrogen and then lyophilized using a vacuum freeze dryer to obtain dried samples. Subsequently, the samples were mixed with KBr and pressed into discs for measurement. UV/vis absorbance was measured using a UV-1650PC spectrophotometer (Shimadzu, Japan), and QDs in deionized water or collagen+genipin solution were prepared and measured after cross-linking for 0 and 24 h.

Nanocomposite scaffold fabrication

Collagen and genipin solutions were prepared prior to the fabrications of different scaffolds. Collagen type I was extracted from calf skin and purified as described previously.[1] After sterilizing, lyophilized collagen was immersed and dissolved in 0.5 M acetic acid and the dissolution process was facilitated by stirring on a magnetic stirrer at 4 °C overnight to obtain a stock solution of collagen. A 500 mM genipin solution was prepared by dissolving 11.3 mg of genipin powder (Macklin Biochemical Co. Ltd, Shanghai, China) in 100 μL of 60% alcohol *via* the ultrasonic dissolution method. Prior to use, ice-cold collagen was neutralized by 1 M NaOH with a final concentration of 15 mg/mL, yielding C hydrogels. 1.98 mL ice-cold collagen solution was taken out and transferred to a tube, and afterwards, 20 μL of genipin ethanol solution was added to the collagen solution. To sufficient reaction, the mixture was stirred for 10 min at a low temperature using a mechanical mixer to allow genipin to occupy the amino site of collagen, obtaining liquid CG hydrogel. The CGQ

hydrogels were fabricated by using collagen and QDs as reactants and genipin as a crosslinker. Typically, 20 μL of QDs (1.85 μM, WUHAN JIAYUAN QUANTUMDOTS CO., LTD, Wuhan, China) solution was added into 1.98 mL liquid CG hydrogel, followed by vigorous stirring for 10 min at 4 °C for homogeneous distribution and crosslinking of QDs to obtain the CGQ hydrogel. The resulting three nanocomposite solutions (*i.e.*, C, CG and CGQ) were stored in refrigerator at 4°C to prevent gelation before use.

Absorbance and fluorescence

The collagen and CGQ solutions were placed in 96-well plates (200 μL per well) and incubated at 37°C for gelation. The plates were covered and kept at 100% RH to prevent evaporation. After 0, 0.5, 1, 2, 4, 8, 12, 24, 48 and 72 h of incubation, the absorbance at 595 nm was measured by a microplate reader, and fluorescence measurements were performed with an excitation wavelength at 595 nm and an emission wavelength at 630 nm using a fluorescence microplate reader. To avoid changes in fluorescence caused by temperature variation, the plates were cooled at room temperature for 20 min.

Swelling and degradation behavior

The collagen, CG and CGQ hydrogels were placed in 96-well plates (200 μL per well) and incubated at 37°C for gelation. The swelling ratio was assessed by using the gravimetric method.[2] Dry specimens (initial weights of W_0) were immersed in PBS (pH 7.4) with gentle shaking at 20°C. At each time point, the swelling samples were weighted (W_{sw}), and the formula $E_{sw} = 100\% \times [(W_{sw} - W_0) / W_0]$ (n=6) was applied to calculate the swelling ratio.

The *in vitro* degradation of a known dry weight (W_0) composite was analyzed in 2 mL of PBS (pH 7.4) containing 25 U of collagenase type I at 37 °C for 24 h (collagenase type I concentration: 100 μg/mL). At each time point, the final dry weight (W_t) of the freeze-dried samples was recorded. The equation $D = 100\% \times (W_0 - W_t) / W_0$ was used for the calculation of the *in vitro* degradation rate of gels. Additionally, another concentration (*i.e.*, 10 ng/mL) of collagenase type I that was approximately identical to that in serum *in vivo* was also used to explore the *in vitro* degradation behaviors of the three hydrogels at 37 °C.

Mechanical test

The mechanical properties of the collagen, CG and CGQ gels were tested using a mechanical tester (Hengyi, Shanghai, China). Scaffold specimens were engineered into cylindrical shape

at a size of 8 mm × 4 mm (*i.e.*, diameter × height) and then proceeded to cross-link at 37 °C for 24 h. The compress dynamics of the gels were detected with a cross head speed of 0.1 mm/min. A 50 N load cell was used and the experiments were repeated in triplicate (n=3). The compressive modulus data were obtained from the linear region (*i.e.*, 10% -20%) of the stress-strain curve.

Photodynamic Provocation (PDP)

A near-infrared laser excited by a Fiber Coupler laser system was used in all experiments. Samples treated with various energy densities (fluence, J/cm^2) were achieved by varying the power density (irradiance, W/cm^2). The optimal irradiation time and energy densities for *in vitro* and *in vivo* studies were determined according to the results of ROS production and cell viability. Cells and animals in the C+PDP, CG+PDT and CGQ+PDP groups received PDP every other day.

Detection of singlet oxygen

The chemical probe 1, 3-diphenylisobenzofuran was employed to measure the singlet oxygen generated by nanocomposites, and collagen served as a control. Typically, 20 μL of ethanol solution containing 10 mM DPBF was added to 2 mL of PBS. Then, the scaffolds were immersed in the above solution and irradiated with an 808 nm laser for 10 min. After centrifugation, the absorbance of DPBF at 410 nm was recorded at each time point using a microplate reader.

Cell culture and seeding in hydrogels

Bone marrow mesenchymal stem cells (BMSCs) were harvested from the femoral marrow of 5-day-old Sprague-Dawley (SD) rats under sterile conditions. The neonatal SD rats were sacrificed by injection of an overdose of sodium pentobarbital (Sigma-Aldrich, USA). Aspirates harvested from bilateral femoral marrow cavities were centrifuged and cultured with complete culture medium comprising α-modified Eagle's medium supplemented with 10% fetal bovine serum and 1% penicillin/streptomycin antibiotics.

For the *in vitro* study, BMSCs at passage 3 were digested and then collected by centrifugation at 1000 rpm for 5 min. Then the cells at a density of 1.0×10^6 cells were seeded into each ice-cold hydrogel solution (100 μL) prior to gelation. The mixture of cells and hydrogel was immediately stirred evenly and then cast in a round mold (0.5 cm in diameter

and 0.5 cm in depth) followed by incubation at 37 °C for gelation (10 min). Subsequently, the BMSC-encapsulated constructs were taken out from the mold and washed with PBS to remove uncrosslinked QDs. The cell/scaffold constructs were cultured in the presence of chondrogenic medium (Cyagen Biosciences Inc. USA) for 7, 14 and 21 days, respectively. The groups were divided as follows: (1) collagen (C); (2) collagen + genipin (CG); (3) collagen + PDP (C+PDP); (4) collagen + genipin + QDs (CGQ); (5) collagen + genipin + PDP (CG+ PDP) and (6) CGQ + photodynamic therapy (CGQ+PDP). For *in vivo* study, injectable cell-scaffold composites were directly used to fill the defect locations of articular cartilage for 4 and 8 weeks. BMSCs cultured with chondrocyte differentiation medium for 14 days and then collected and mixed with scaffolds as described above were subcutaneously implanted into nude mice.

Intracellular ROS detection

The intracellular ROS production in four hydrogels *in vitro* and *in vivo* was measured by a dihydroethidium probe kit. After being rinsed three times with serum-free α-MEM, the hydrogels with seeded cells were preloaded with DHE probe and irradiated using an 808 nm laser. NAC (a ROS scavenger) and EAtB were used as controls. The fluorescence in cells was visualized and photographed using a confocal laser microscope.

CCK-8 assay

The cytotoxicity of laser-irradiated cells in the composites was determined with a Cell Counting Kit-8. Briefly, cells encapsulated in the CGQ hydrogel at 72 h post laser irradiation were incubated with 100 µL of fresh cell culture medium containing 10 µL of CCK-8 at 37°C for 3 h. The samples were crushed to pieces and then centrifuged. The absorbance of the supernatant at 450 nm was analyzed by using a microplate reader.

Cell viability

A live/dead cell staining kit was used to assess the viability of BMSCs cultured in scaffolds *in vitro* at the endpoint (21 days). Cells were rinsed three times with PBS and then treated with 2 µM calcein-AM and 4 µM propidium iodide (PI) in medium minus FBS at room temperature and protected from light for 5 min. Then, the sample was rinsed three times with PBS and photographed with a confocal laser scanning microscope (CLSM) with 494 emission and 528 nm excitation wavelengths.

F-actin cytoskeleton observation

Cells seeded on the scaffolds and cultured for 21 days were rinsed using PBS and fixed in 4% paraformaldehyde solution for 30 min. Subsequently, the cells were permeabilized with 0.1% Triton X-100 for 5 min and stained with rhodamine-conjugated phalloidin at a dilution of 1:1000 in PBS for 30 min in the dark. After washing three times with PBS, 4′, 6-diamidino-2-phenylindole (DAPI, 1:500) was used to stain the nuclei. The cells were then visualized and digitally scanned using a CLSM.

Biochemical assays

At each time point, samples were rinsed in PBS, minced and digested with 500 μL of proteinase K solution (50 μg/mL) at 60°C for 16 h for quantification of DNA and glycosaminoglycan (GAG). DNA contents were measured using the Hoechst 33258 dye with DNA from the calf thymus as a standard. The GAG contents were quantified spectrophotometrically using dimethylmethylene blue chloride methods. The synthesis of GAG was quantified with a calibration curve of chondroitin sulfate absorbance and standardized to the total DNA contents of the constructs.

Quantitative real time polymerase chain reaction (qRT-PCR) analysis

Gene expression levels of cells seeded into collagen, CG and CGQ scaffolds with or without PDP after 7, 14 and 21 days of culture were analyzed by qRT-PCR. A Quanti Tect Reverse Transcription kit was used for reverse transcription (RT) of total RNA to obtain cDNA. qRT-PCR was performed using Fast Start Universal SYBR Green Master Mix on a detection system. The cycling conditions for PCR were 95°C for 3 min, followed by 40 cycles of 95°C for 3 s and then 30 s at 60°C. The forward and reverse primer sequences for *Acan, Sox9, Col2a1, Col1a1* and *β-actin* are listed in **Table S1**. The mRNA expression levels were quantified using *β-actin* as an internal control and calculated based on the threshold cycle (Ct) method as $R= 2^{-\Delta\Delta CT}$.

Animal procedure

In total, sixty female nude mice (aged 6-8 weeks, 15-18 g) and fifty female SD rats (8-10 weeks of age, 200-220 g) were used in the present study. Animal experiments were conducted in compliance with the Guidelines for Animal Experimentation. Nude mice were administered with a subcutaneous implant containing 200 μL of cell-scaffold composites for 4 and 8 weeks.

The body weight was measured twice a week. After general anesthesia with 30 mg/kg of 2% sodium pentobarbital, a cartilage-only defect (2 mm in diameter × 1.5 mm in depth) was generated in SD rats in the center of each patellar groove. Then, 50 μL of cell-scaffold composites described above was implanted into the defect for 4 and 8 weeks of treatment.

***In vivo* ROS detection after PDP**

ROS generation in constructs that were subcutaneously implanted in nude mice was detected by using a probe kit with nonfluorescent compound DCFH-DA. In brief, hydrogels preloaded with the DCFH-DA probe with encapsulated monolayer cultured cells were injected subcutaneously into the mice. Subsequently, the mice received PDP using the optimal in vitro laser fluence. The fluorescence images were acquired with λ_{ex} = 488 nm and λ_{em} = 525 nm on an In-vivo Image System under anesthesia. Fluorescence signal intensity was quantified as the average of all detected photon counts according to the region of interest. For the treatment of cartilage defects, the injected CGQ constructs in the defects of joints were harvested after irradiation with 808 nm laser at power densities of 0, 1.67, 5.60, 16.7, 22.2 and 27.8 mW/cm^2 for 3 min and the cytotoxicity was analyzed in accordance with the *in vitro* study to determine the optimal laser fluence.

Gross observation

The subcutaneous constructs of nude mice and the repaired articular cartilage of the femoral condyle of SD rats were collected for macroscopic observation and photographed at 4 and 8 weeks after treatment. Three independent observers who were blinded to the experimental groups performed the morphological evaluation of cartilage regeneration according to the O´Driscoll scoring system.[3]

Biomechanical tests

After macroscopic evaluation, a mechanical tester was used to analyze the equilibrium modulus of the repaired articular cartilage at 4 and 8 weeks post treatment. The engineered cartilage was mounted on the testing machine, and the biomechanical stiffness was assessed after all parameters were set. The speed of the cross-head was 0.01 mm/min, and the ratio of equilibrium force to cross-sectional area was divided by the applied strain to calculate the equilibrium modulus (unit: MPa).

Histological and immunohistochemical analysis

Specimens from nude mice and SD rats were fixed with 4% neutral buffered formalin for 48 h. Then, femoral condyles from SD rats were decalcified in aqueous solution (pH 7.2) containing 14% (w/v) EDTA and 10% (v/v) ammonium hydroxide with an ultrasound machine for 2 weeks. After dehydration by graded ethanol, the constructs were paraffin-embedded and cut into 3 mm slices. Cells were stained with hematoxylin-eosin and safranin O/fast green. The expression of COL2A1 was analyzed by immunohistochemical staining. Primary antibodies against COL2A1 and the Biotin-Streptavidin HRP Detection System were used. The staining was developed with DAB solution and counterstained with hematoxylin. Pictures were obtained using a phase contrast microscope. The repaired articular cartilage tissues in each group were graded using the ICRS Visual Histological Assessment Scale by three blinded independent observers.

Western blot analysis

Cells seeded in the scaffolds were harvested and lysed with RIPA buffer with the addition of phenylmethanesulfonyl fluoride, a protease and phosphatase inhibitor. Cell lysates were incubated on ice for 3 h and then the supernatant was collected after centrifugation. A 10% SDS-PAGE gel was used for electrophoresis with a total of 60 μg of protein loaded in each lane. The target proteins on the SDS-PAGE gel were transferred to polyvinylidene fluoride membranes (PVDF; 0.45 μm), followed by blocking with 5% blocking buffer at 37°C for 1 h. PVDF membranes were treated with primary antibodies against COL2A1, SMAD2/3, phosphorylated-SMAD2/3, mTOR, phosphorylated-mTOR, and β-actin at 4°C overnight. Subsequently, the membranes were rinsed with TBST three times and incubated with the secondary antibody for 1 h. An imaging system was used to scan the membranes, and densitometry of the bands was performed with Image 2x.

Statistical analysis

One-way ANOVA followed by an LSD post hoc test was performed to calculate the statistical significance of differences between the groups by using the SPSS 22.0 (SPSS Inc., Chicago, USA). All data were collected from triplicate samples and are presented as the mean ±standard deviation (SD), with the significance of differences between groups set such that p-values < 0.05 were considered to indicate statistically significant differences.

References

[1] a) Z. Lu, D. Lei, T. Jiang, L. Yang, L. Zheng, J. Zhao, *Cell Death Dis.* **2017**, *8*, e2801; b) T. Jiang, G. Xu, Q. Wang, L. Yang, L. Zheng, J. Zhao, X. Zhang, *Cell Death Dis.* **2017**, *8*, e2851.

[2] L. Michalek, L. Barner, C. Barner-Kowollik, *Adv. Mater.* **2018**, *30*, e1706321.

[3] V. M. Betz, A. Keller, P. Foehr, C. Thirion, M. Salomon, S. Rammelt, H. Zwipp, R. Burgkart, V. Jansson, P. E. Muller, O. B. Betz, *J. Gene Med.* **2017**, *19*, e2972.

Supplementary figures

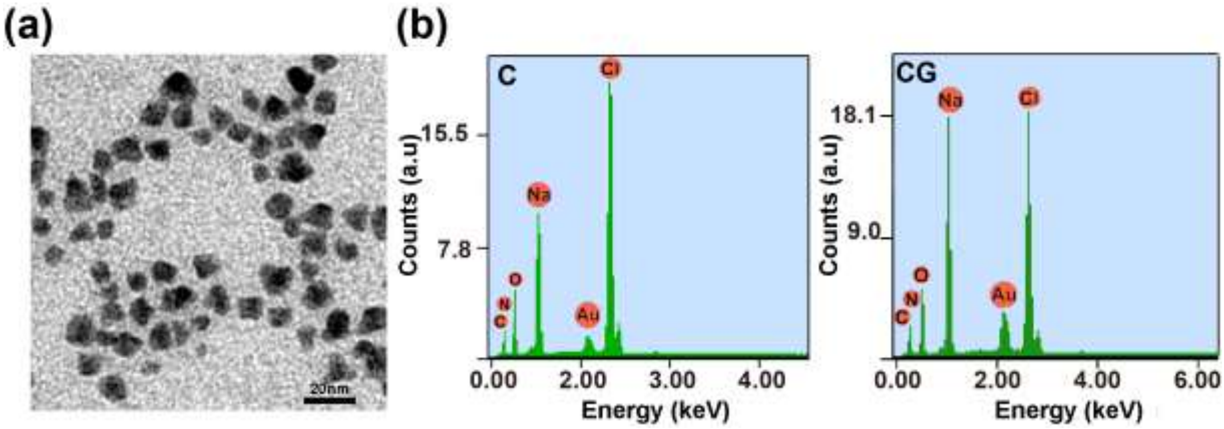

Figure S1 (a) TEM image of CdSe QDs in ethanol and (b) EDS spectra of the C and CG hydrogels (C=collagen, CG= collagen crosslinked with genipin).

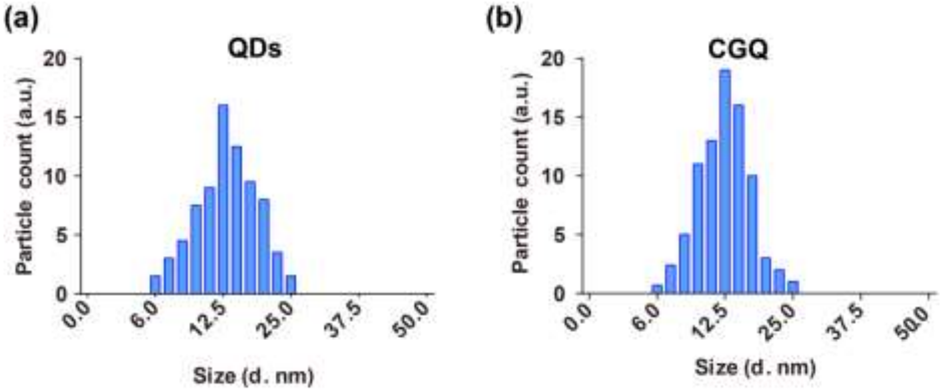

Figure S2 Size distributions of QDs in water solution (a) and liquid CGQ hydrogel (b) (CGQ = collagen crosslinked with genipin and QDs).

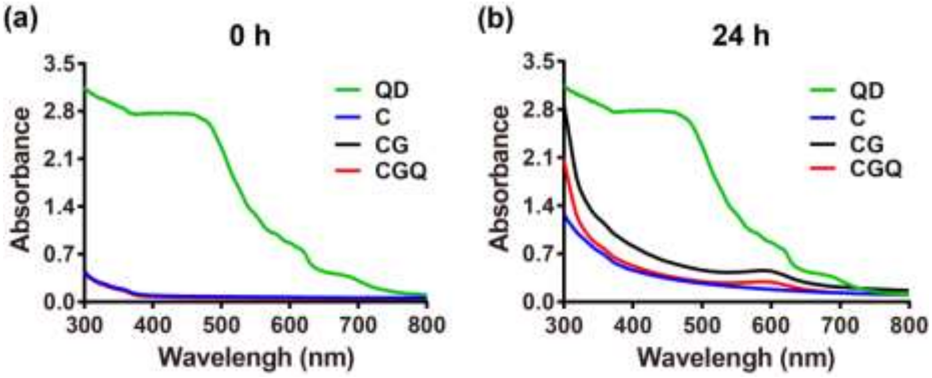

Figure S3 (a) Ultraviolet absorption spectra of QDs, C, CG and CGQ solutions at 0h. **(b)** Ultraviolet absorption spectra of QDs, C, CG and CGQ after incubation at 37 ℃ for 24 h (C=collagen, CG=collagen crosslinked with genipin, CGQ=collagen crosslinked with genipin and QDs).

Table S1 The initial gelation point of collagen-based hydrogels at 4 °C and 37 °C.

Scaffolds	Gel point (h) at 4 °C	Gel point (s) at 37 °C
C	30.5±2. 1	456±30
CG	26.4±2.7	433±34
CGQ	22.9±3.3	421±20

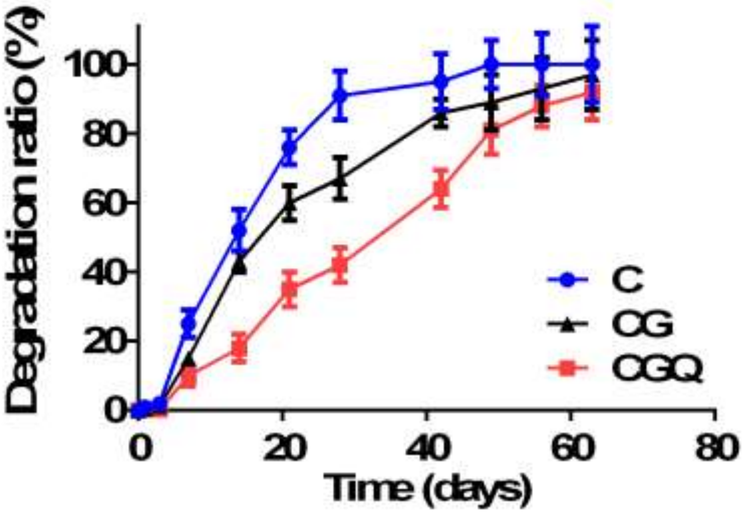

Figure S4 Time-dependent degradation ratios of C, CG and CGQ hydrogels at a type I collagenase concentration of 10 ng/mL.

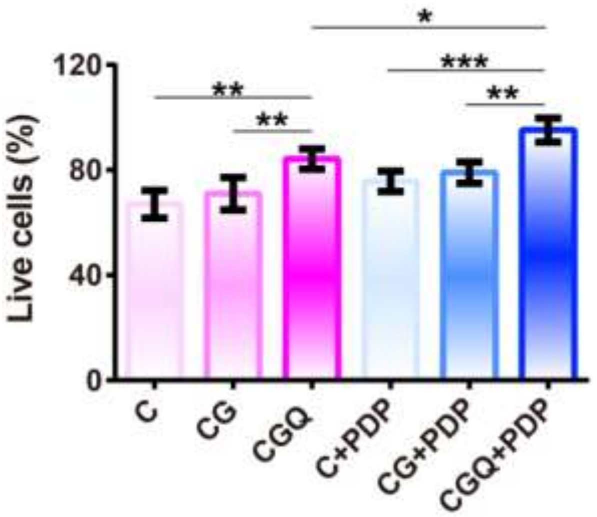

Figure S5 Percentages of live cells determined from the plots in **Figure 4d**. Mean ±SD, n=5; * indicate $p < 0.05$, ** indicate $p < 0.01$, *** indicate $p < 0.001$ (C=collagen + BMSCs, CGQ=collagen crosslinked with genipin and QDs + BMSCs, C+PDP=collagen + BMSCs+ irradiation with an 808 nm laser at fluence of 3 J/cm^2 for 3 min, CGQ+PDP= CGQ scaffold +BMSCs + irradiation with an 808 nm laser at fluence of 3 J/cm^2 for 3 min).

Table S2 The forward and reverse primer sequences of different genes for RT-PCR performance**.**

Gene name	Forward primer	Reverse primer
Acan	5'- CCGCTGGTCTGATGGACACT-3'	5'- AGGTGTTGGGGTCTGTGCAA-3'
Col2a1	5'- CTGGTCCTTCCGGCCCTAGA-3'	5'- GGATCGGGGCCCTTCTCTCT-3'
Sox9	5'-TCCAGCAAGAACAAGCCACA-3'	5'- CGAAGGGTCTCTTCTCGCTC-3'
Col1a1	5'- CATGAGCCGAAGCTAACCC-3'	5'- CTCCTATGACTTCTGCGTCTGG-3'
β-actin	5'-CCCATCTATGAGGGTTACGC-3'	5'-TTTAATGTCACGCACGATTTC-3'

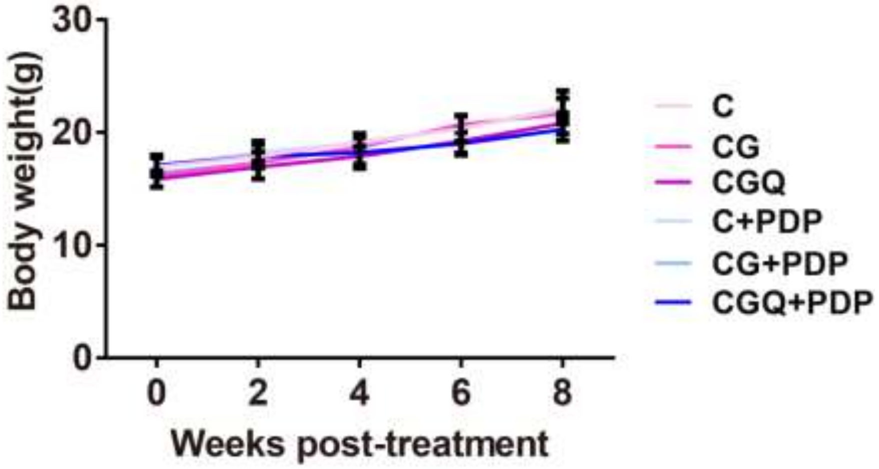

Figure S6 Body weight changes of nude mice that received different treatments (C=collagen + BMSCs, CG=collagen crosslinked with genipin + BMSCs, CGQ=collagen crosslinked with genipin and QDs + BMSCs, C+PDP=collagen + BMSCs + irradiation with an 808 nm laser at fluence of 3 J/cm^2 for 3 min, CG+PDP=CG scaffold + BMSCs + irradiation with an 808 nm laser at fluence of 3 J/cm^2 for 3 min, CGQ+PDP= CGQ scaffold + BMSCs + irradiation with an 808 nm laser at fluence of 3 J/cm^2 for 3 min).

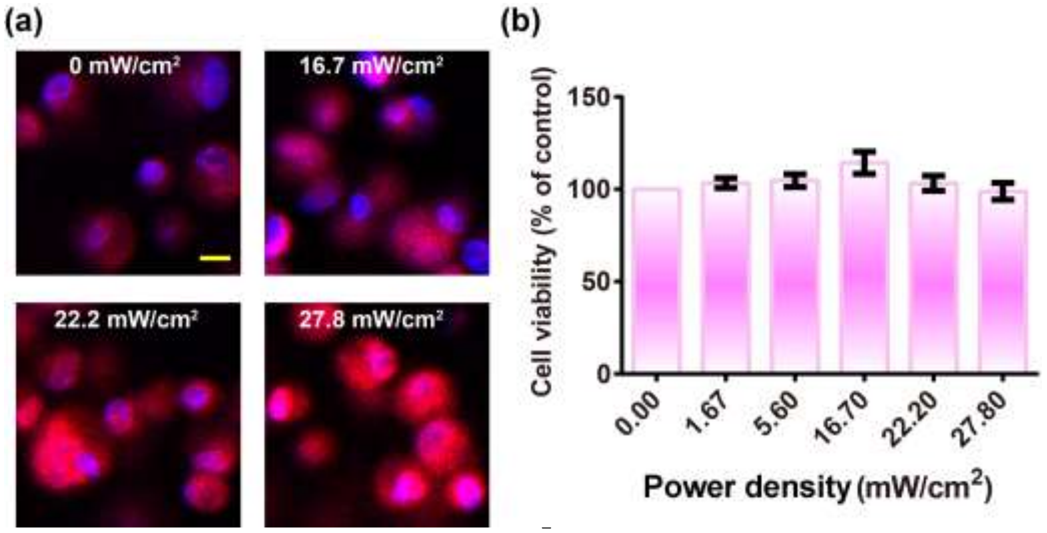

Figure S7 Determination of optimal irradiation fluence for PDP in joints. (a,b) LCSM images of ROS generation (a) and cell viability (b) of the CGQ constructs harvested from cartilage defects after irradiation at different power densities for 3 min (scale bar: 20 μm).

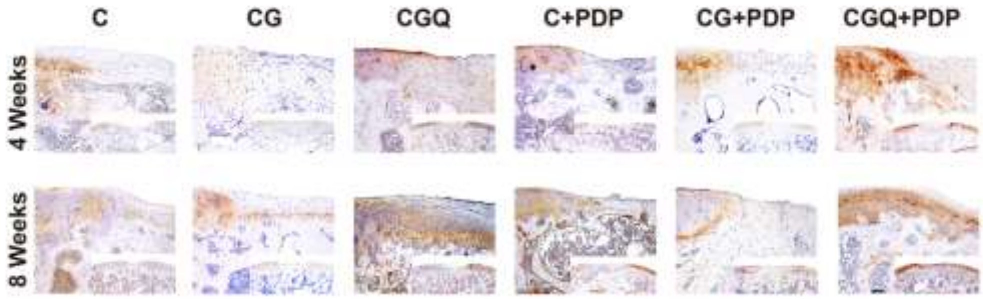

Figure S8 Immunohistochemical staining for collagen type II in the engineered cartilage tissues, sale bar=200 μm (C=collagen + BMSCs, CG=collagen crosslinked with genipin + BMSCs, CGQ=collagen crosslinked with genipin and QDs + BMSCs, C+PDP=collagen + BMSCs + irradiation with an 808 nm laser at fluence of 3 J/cm^2 for 3 min, CG+PDP= CG scaffold + BMSCs + irradiation with an 808 nm laser at fluence of 3 J/cm^2 for 3 min, CGQ+PDP= CGQ scaffold + BMSCs + irradiation with an 808 nm laser at fluence of 3 J/cm^2 for 3 min).

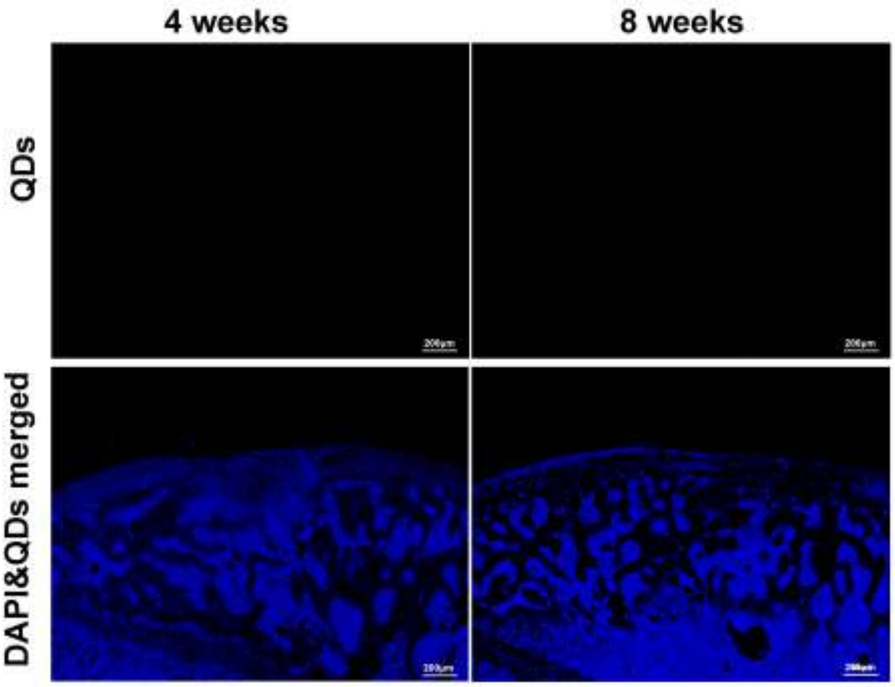

Figure S9 LCSM images of regenerated cartilage tissues after treatment with CGQ+PDP for 4 weeks and 8 weeks, respectively, and the excitation wavelengths of CdSe QDs and DAPI staining for cell nuclei are 605 nm and 454 nm, respectively.

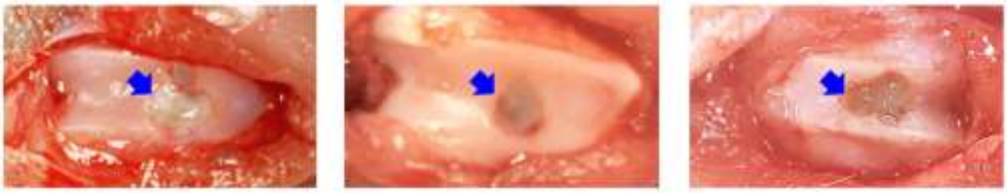

Figure S10 Digital photos of defects with varied shapes wherein injectable CGQ hydrogels were engineered into various shapes for cartilage repair.
